# Funding illness prevention and health promotion in Australia: a way forward

**DOI:** 10.1186/1743-8462-6-25

**Published:** 2009-11-12

**Authors:** Anthony Harris, Duncan Mortimer

**Affiliations:** 1 Centre for Health Economics, Monash University, Clayton, Victoria, 3800, Australia

## Abstract

**Background:**

Unlike pharmaceuticals and private medical services there is no single source of funding for illness prevention and health promotion and no systematic process for setting priorities in public health. There is a need to improve the efficiency of access to health funding across prevention and treatment.

**Discussion:**

We discuss a number of reforms to existing funding arrangements including the creation of a national Preventative Priorities Advisory Committee (PrePAC) to set priorities. We propose the establishment of a PrePAC to provide evidence and set priorities across health promotion and illness prevention, with a national dedicated fund for health promotion.

**Conclusion:**

A national evidence-based funding system for illness prevention and health promotion would legitimise a substantial and sustained budget for health promotion, breaking down some of the barriers in a fragmented federal health care system.

## Background

Funding for illness prevention and health promotion in Australia is fragmented. Programs are funded by federal, state and local government but a lack of coordination and priority setting means that we are not making a considered allocation of resources to illness prevention and health promotion. We neither choose how much to spend compared to other health care activities, nor do we choose interventions that offer the best value. Australia has led the world in introducing formal evidence-based evaluation processes for medical services and technologies to establish value for money, but, as a consequence of fragmented responsibility and funding, health promotion and illness prevention have been left out. While pharmaceuticals and (out of hospital and private hospital) medical services in Australia have access to dedicated budgets and are subject to formal and well-defined evaluation processes, most prevention and health promotion interventions compete for disparate and uncertain sources of funding that lack consistent and rational criteria for allocation. In Australia, differences in the likelihood of funding among otherwise equivalent interventions can be attributed partly to differences in funding arrangements [1]. An efficient health care system would not discriminate in this way. In this paper we suggest the creation of a national Preventative Priorities Advisory Committee (PrePAC) to reduce the gap in the likelihood of obtaining public funding among equally cost effective interventions.

## Discussion

### Two Models for a PrePAC

Two stylised models for the operation of a PrePAC are considered here: a (i) Guidance Model and a (ii) Dedicated Funding Model.

### Guidance model

The UK National Institute of Clinical Excellence (NICE) provides evidence-based guidance relating to health technologies and public health interventions [2-4]. The Public Health Interventions Advisory Committee (PHIAC) and Technology Appraisal Committees of NICE produce recommendations on the use of public health interventions and health technologies--based on consideration of the available evidence regarding effectiveness and the cost effectiveness. Under the guidance model, a proposed PrePAC would take a similar role to that of the PHIAC in England. It would take account of the strength of evidence on effectiveness and cost effectiveness of a prevention activity and might, for example, provide guidance that a particular activity should be given higher or lower priority. While the quality of evidence in the area of prevention is often not high, this is not necessarily a barrier to good decision-making [5]. It would produce a list of recommendations that funding agencies could take into account when making funding decisions and setting priorities for prevention. In 2009, COAG agreed to fund such a national preventive health agency with responsibility for providing evidence-based policy advice [6]; this model was endorsed by both the National Health and Hospital Reform Commission [7] and the Preventative Health Taskforce [8].

The main problem with a guidance model is that there is no mechanism for funding. The guidance model relies on a disparate set of independent funding agencies to implement PrePAC deliberations and recommendations. While the various responsible funding agencies might modify their own funding rules or processes to take account of PrePAC deliberations and recommendations, such recommendations would be neither necessary nor sufficient to secure funding. There may be no new funding allocated for prevention and no guarantee that the deliberations and recommendations of a PrePAC will have any impact at all upon resource allocation. Implementation involves much more than simply developing and disseminating guidance. The Guidance model therefore falls well-short of giving preventative interventions an equal chance of funding compared to otherwise equivalent interventions that benefit from access to a well-defined funding mechanism.

### Dedicated Prevention Fund

A PrePAC allocating a dedicated prevention fund could be modelled on the operation of the Medical Services Advisory Committee (MSAC) [9] or the Pharmaceutical Benefits Advisory Committee (PBAC) in Australia [10]. Specifically, a PrePAC would advise a fund-holder on whether a particular intervention or program should be allocated money from a dedicated prevention fund. Recommendations accepted by the fund-holder would then be listed on a Prevention Benefits Schedule (PreBS). Depending on funding arrangements, the fund-holder might be one or more of the Australian Health Ministers. For clinical prevention services provided by a registered provider to an individual patient, the PreBS could operate in much the same way as individual patient benefits listed on the Medicare Benefits Schedule (MBS) or the Pharmaceutical Benefits Schedule (PBS). That is to say, there would be a list of interventions approved for funding from the scheme on a per-service (e.g. individual consultation on diet or smoking behaviour) or per-patient basis (e.g. annual enrolment in a disease management program).

Following Goldsmith et al. [11], we distinguish clinical prevention from non-clinical preventative interventions such as: (i) health promotion that targets a population to encourage healthy behaviour and is provided by government or interest group organisations, (ii) health protection that reduces health risk by changing the physical or social environment often by regulation, and (iii) healthy public policy that involves social or economic interventions beyond the health sector. Individual patient benefits may be unsuitable for funding these categories of non-clinical prevention and alternative mechanisms would need to be used. For example, payments for health promotion interventions might be based on a list of lump-sum grants to cover a delimited set of activities over a defined time period. Examples might include: annual budget allocation to organisation(s) responsible for delivery of mass media education campaigns on road safety, healthy eating, physical activity, alcohol consumption, or smoking cessation; lump sum payment to local councils or community organisations for community events to promote community connectedness, safe communities or healthy lifestyle; or lump sum payment to schools for the establishment of programs to promote healthy lifestyle such as the walking school bus or sex education.

For healthy public policy and health protection interventions, the PreBS could be a list of lump-sum grants to cover a delimited set of activities at a specific geographic location. Examples might include: lump sum payment to local councils, state governments or community organisations to alter the physical environment either to promote healthy lifestyle (such as the provision of walking tracks/pedestrian crossings, or improvements to parks and recreation areas) or to improve safety (provision of street signs near school crossings or provision of lighting or CCTV around train stations or shopping precincts); lump sum payment to schools to alter the physical environment to promote healthy lifestyle (such as establishment of a kitchen garden or sporting facilities); lump sum payment to schools to alter the social environment to improve safety (such as provision of interventions to reduce bullying).

Reimbursement via lump sum payments and annual budget allocations is no different in principle than per-service or per-patient payments. In each case evaluation of the safety, effectiveness and cost effectiveness of all preventative interventions would be for an approved indication and scale. For clinical prevention, the indication would typically relate to characteristics of the patient and the provider. For example, provision of group exercise sessions might only provide good value for money in people with diabetes or cardiovascular disease if they are predominantly focused on cardiovascular fitness (rather than strength or flexibility training). For health promotion and health protection interventions, the indication might instead relate to characteristics of the community. Provision of CCTV around train stations might only provide good value for money in areas that have a high level of street crime.

The PrePAC might therefore approve open-ended funding for a listed item in much the same way as the PBAC or MSAC, specifying an approved indication for each item. For clinical prevention, claims for PreBS items might follow similar procedures as for restricted benefit items on the PBS. The PrePAC might, however, choose to specify "pre-approval" status for some types of interventions; particularly for health promotion and health protection interventions that entail relatively large lump sum payments. Obtaining approval might require submission of evidence to substantiate that the intended use of funds meets the restriction for the relevant item number. For example, a council requesting funding for a pedestrian crossing might be required to provide traffic impact analyses, a community survey demonstrating community support, and an assessment of the impact of community structure on access to community nodes. Provision of funding for a specific pedestrian crossing with authority required status would therefore entail a two-stage process. First, the PrePAC would have to list pedestrian crossings on the schedule for an approved 'indication'. Second, the local council requesting funding for a specific pedestrian crossing would have to submit a request for approval demonstrating that the intended site meets the 'approved indication' and that the intervention conforms with the approved description in terms of its active constituent parts delivered effectively.

While the dedicated fund model has the advantage of simplicity and familiarity it does have a few boundary issues that would need to be resolved. Regulatory or infrastructure interventions may not sit easily in a health system funding scheme. Smoking or alcohol regulation or the provision of public transport could be appraised by a PrePAC but it is more difficult to imagine a PreBS with the capability to facilitate legislative change or to provide the necessary funding incentives for major infrastructure developments.

### A proposal for reform

Currently clinical prevention is partially covered under existing programs (such as the MBS, PBS, and NIP). Other prevention and health promotion activities are more or less covered by one of a multiplicity of government (federal, state and local) and non-government organisations (such as the National Heart Foundation and Cancer Councils). Health protection and healthy public policy are currently handled within the relevant departments of federal, state or local governments. We propose reforms to existing funding arrangements that take account of the current state of play while increasing the efficiency of access to health funding across prevention and treatment. Specifically, we propose a PrePAC providing guidance with regard to health promotion, health protection and healthy public policy, but that relies on existing programs (such as the MBS, PBS, and NIP) to provide guidance and funding for clinical prevention. Delimiting the scope of a PrePAC in this way would capture the majority of interventions while minimising the risk of replicating the function of existing agencies. Gaps in the coverage of clinical prevention would then be handled by modifying the scope of services covered under existing programs by, for example, issuing Medicare Provider Numbers to a broader range of health care providers and adding item numbers to the MBS. Given the multiplicity of parties currently involved in appraisal of health promotion, health protection and healthy public policy (in contrast to the relatively small number of agencies with responsibility for clinical prevention), the creation of a PrePAC would yield substantial cost savings and improvements in the consistency and quality of evidence appraisals.

For health promotion, we believe that the PrePAC should be extended beyond the guidance model to allocate a national fund for health promotion activities. The creation of a PrePAC with responsibility for funding health promotion has the potential to reduce the number of programs making allocation decisions and can be expected to improve the consistency and quality of decision-making. A PrePBS would provide national coverage of health promotion activities and a clear pathway for funding for interventions that currently have no obvious source of funding.

While we believe that a PrePAC could make a valuable contribution by providing guidance for non-clinical prevention, many preventative interventions that might be categorised as health protection and healthy public policy would not be amenable to funding through an item based PreBS-type mechanism. The large-scale inter-sectoral nature of some health protection and healthy public policy interventions (such as transport and infrastructure) and the non-financial costs of implementing of others (such as smoking restrictions) are not easily achieved through the allocation of a dedicated fund. For this reason, we propose that the PrePAC should initially provide guidance only for health protection and healthy public policy.

## Conclusion

We agree with the Wanless report [12] that "to achieve the objective of an efficient allocation of national health service funding between health care and public health, a similar method of cost effectiveness analysis needs to be applied to public health and clinical interventions." Our suggestion is to create a new body--the Prevention Priorities Advisory Committee (PrePAC). The PrePAC would provide advice on the effectiveness and cost effectiveness of interventions in health protection and healthy public policy. This is consistent with aspects of other proposals to establish a nationally coordinated preventive health agency responsible for the evaluation of effectiveness, efficiency and equity outcomes [6,8,13]. It has the potential to engage many of the levers identified by Lin [14] for shifting the focus of the health system to more emphasis on prevention and health promotion. We believe that it is necessary to go further and establish a national dedicated fund for health promotion, access to which would be determined by the PrePAC. This would provide a national program, thus breaking down some of the barriers in a fragmented federal health care system. It would provide technical leadership and a political voice for prevention that has been lacking compared to medical services and pharmaceuticals. Perhaps most importantly it would legitimise a substantial sustained budget for health promotion that would be directly translated into policy choices. In doing so it has the potential to improve the efficiency and equity of the overall health care system.

## Competing interests

This paper is based on an options paper commissioned by the National Health and Hospitals Reform Commission [5].

## Authors' contributions

AH and DM conceived the article. AH reviewed and edited the draft prepared by DM. Both authors read and approved the final manuscript.
